# Intra- and Inter-Observer Variability of Quantitative Parameters Used in Contrast-Enhanced Ultrasound of Kidneys of Healthy Cats

**DOI:** 10.3390/ani12243557

**Published:** 2022-12-15

**Authors:** Amber Hillaert, Emmelie Stock, Sophie Favril, Luc Duchateau, Jimmy H. Saunders, Katrien Vanderperren

**Affiliations:** 1 Department of Morphology, Imaging, Orthopedics, Rehabilitation and Nutrition, Faculty of Veterinary Medicine, Ghent University, 9820 Merelbeke, Belgium; 2 Small Animal Department, Faculty of Veterinary Medicine, Ghent University, 9820 Merelbeke, Belgium; 3 Cancer Research Institute Ghent, 9000 Ghent, Belgium; 4 Department of Comparative Physiology and Biometry, Faculty of Veterinary Medicine, Ghent University, 9820 Merelbeke, Belgium

**Keywords:** contrast-enhanced ultrasound, observer variability, kidney, cat, feline

## Abstract

**Simple Summary:**

Contrast-enhanced ultrasound is a technique which enables assessment of tissue blood flow by injecting gas-filled micro-bubbles in the bloodstream. As a diagnostic and monitoring tool, this technique has shown great promise in multiple clinical conditions, while offering many advantages over other imaging techniques. A major challenge with this technique, however, is the fact that the imaging results can vary significantly, and there is no comprehensive understanding of their cause. Improving the understanding of the sources of variation could contribute to the development of guidelines to reduce variation and promote the application of CEUS in practice. The aim of this study was to assess variability in blood flow measurements in the kidney due to observers. As regions of interests are manually delineated using a specialized software, positioning of the regions may differ between observers and within the same observer at different occasions. This study found that a number of evaluated parameters had a good inter- and intra-observer agreement, while other parameters had a considerably lower inter-observer agreement compared to intra-observer-agreement. Therefore, it may be advisable that quantitative assessment of renal perfusion is not carried out by different researchers, especially if their experience levels differ.

**Abstract:**

Contrast-enhanced ultrasound (CEUS) is a non-invasive imaging technique which allows qualitative and quantitative assessment of tissue perfusion. Although CEUS offers numerous advantages, a major challenge remains the variability in tissue perfusion quantification. This study aimed to assess intra- and inter-observer variability for quantification of renal perfusion. Two observers with different levels of expertise performed a quantitative analysis of 36 renal CEUS studies, twice. The CEUS data were collected from 12 healthy cats at 3 different time points with a 7-day interval. The inter- and intra-observer agreement was assessed by the intraclass correlation coefficient. Within and between observers, a good agreement was demonstrated for intensity-related parameters in the cortex, medulla, and interlobular artery. For some parameters, ICC_inter_ was considerably lower than ICC_intra_, mostly when the ROI encompassed the entire kidney or medulla. With the exception of time to peak (TTP) and mean transit time (mTTI), time-related and slope-related parameters showed poor agreement among observers. In conclusion, it may be advised against having the quantitative assessment of renal perfusion performed by different observers, especially if their experience levels differ. The cortical mTTI seemed to be the most appropriate parameter as it showed a favorable inter-observer agreement and inter-period agreement.

## 1. Introduction

Contrast-enhanced ultrasound (CEUS) is a non-invasive imaging technique which allows qualitative and quantitative assessment of tissue perfusion at the macro- and microvascular level [1]. This imaging technique is based on the detection of harmonic signals reflected by encapsulated micro-bubbles in the bloodstream during exposure to ultrasound waves [2,3]. Over the past decades, the number of clinical applications of CEUS has increased considerably [1,3,4,5,6]. These include, among others, diagnostic work-up of lesions in various organs, evaluation of treatment efficacy, echocardiography, follow-up of renal transplants, and guidance of biopsy [1,3,4,5,6]. Compared with other imaging modalities for evaluating vascular perfusion, such as computed tomography (CT) or magnetic resonance (MR), CEUS is considered a more favorable technique due to its excellent safety profile, cost efficiency, lack of ionizing radiation, and lack of renal toxicity [4,7].

In nephrology, CEUS is highly interesting as many renal disorders are characterized by vascular or hemodynamic alterations [4]. These changes occur early in the development of renal dysfunction and sometimes precede clinical signs of renal impairment [2,7]. Therefore, CEUS could allow earlier diagnosis in certain kidney disorders, which would enable earlier therapeutic intervention. In addition, CEUS has been shown to be valuable for assessing the effect of drugs on renal perfusion and has brought new insights into the pathophysiology of renal associated diseases [8,9,10,11]. 

Various studies have assessed the application of CEUS in the evaluation of kidney diseases in human and veterinary medicine. A pilot study by Dong et al. (2014) found encouraging results indicating that CEUS can detect early stages of chronic kidney disease (CKD) in humans [12]. Similar results were obtained in cats, where CEUS was able to detect an altered perfusion in cats with CKD [13]. Studies in dogs demonstrated that acute kidney disease (AKI) gives rise to altered renal perfusion, which could be helpful for diagnosis [10,11]. Furthermore, researchers found that CEUS could predict progression from ischemic AKI to CKD in mice [14].

An important drawback of CEUS, however, is its potential high variability, which may lead to diagnostic uncertainty [1,15]. Within nephrology, the use of CEUS is therefore mainly restricted to research purposes, while translation into clinical practice remains limited. The variability of CEUS has mainly been attributed to patient-related factors (e.g., body temperature, blood pressure, and heart rate), scanner settings (e.g., mechanical index, gain, focus position, and tissue gain compensation), and contrast agent-related factors (e.g., microbubble type, preparation, dosage, and injection method) [15]. By influencing the microbubble concentration and/or properties, these factors can cause variation in bubble echo signals and thus quantification [15]. However, there are other possible sources of variability that have not yet been thoroughly investigated. The process of drawing regions-of-interest (ROIs) on specific areas of the kidney for perfusion quantification, among others, might introduce an additional source of variation. The ROIs may vary to a certain extent in location, depth, and size, factors which have been found to influence the TICs (time intensity curve) and the perfusion parameters derived from them [15,16,17,18,19,20]. 

Improving the understanding of the sources of variation could contribute to the development of guidelines to reduce variation and promote the application of CEUS in practice. Therefore, this study aimed to assess intra- and inter-observer variability for quantification of renal perfusion using a bolus injection of contrast agent.

## 2. Materials and Methods

For this study, movie clips were used from a previously published repeatability study evaluating normal intra- and inter-individual variation in healthy cats [21].

Briefly, twelve healthy European Shorthair cats, 9 female neutered and 3 male neutered cats, were included with the permission of the local ethical committee of Ghent University (EC 2014/148). The age of the cats ranged between 3.92 years and 13.17 years (median, 6 years and 9 months) and their body weight ranged between 2.75 and 3.55 kg (median, 3.28 kg).

At 3 time points with a 7-day interval, CEUS of both feline kidneys was performed. Every time point, a catheter was placed in one of the cephalic veins. Anesthesia was induced with a bolus of propofol (Propovet 10 mg/mL; Abbott Laboratories), 4–8 mg/kg intravenously, given to effect, and maintained with additional boluses (1 mg/kg) as necessary. 

CEUS, using bolus injection of Sonovue (0.05 mL/kg, Bracco, Milan, Italy) was performed as previously described [21]. Briefly, the kidneys were imaged in longitudinal plane by the same person, using a 12–5 MHz linear transducer on a dedicated machine (iU22, Philips, Bothell, WA, USA) with contrast-specific software. Three to four injections of contrast were performed: the first one was not used for further quantification since this injection often results in lower enhancement [22]. Basic technical parameters were held constant across all cats [21]. A low mechanical index of 0.09, dynamic range of 50, gain of 85%, persistency off, and side-by-side imaging were used. A single focus was placed directly under the kidney. Images were recorded at a rate of 7 frames/s for 90 seconds as movie clips. 

The movie clips were exported as DICOM and analyzed using specialized computer software (VueBox^®^ v6.2.0.55291, Bracco Suisse SA, Planles-Ouates, Switzerland) for objective quantitative analysis. The studies were randomly selected, 1 kidney (left or right) was randomly selected at each time point with the only limiting factor that each kidney of each cat was at least evaluated once. The studies were duplicated for intra-observer analysis, giving a total of 72 anonymized studies that were randomly presented to 2 blinded observers with different degrees in experience with the software program after collection of all images. One observer was a board-certified radiologist with 5 years of experience with CEUS (E.S.), the second observer was a PhD student with minimal experience with CEUS (S.F.).

**Figure 1:**
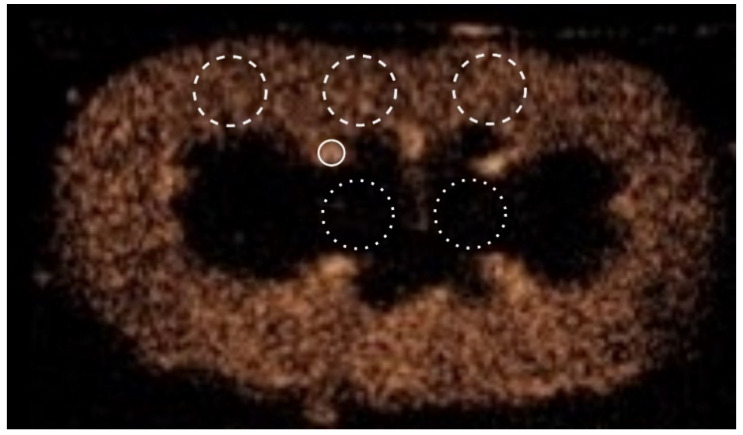
Illustration of the position of the regions-of-interest on a contrast-enhanced ultrasound image of a healthy cat’s kidney. Cortex regions-of-interest are enclosed by a white striped line, medulla regions-of-interest by a white dotted line, and the region-of-interest surrounding the interlobar artery by a solid white line.

**Figure 2:**
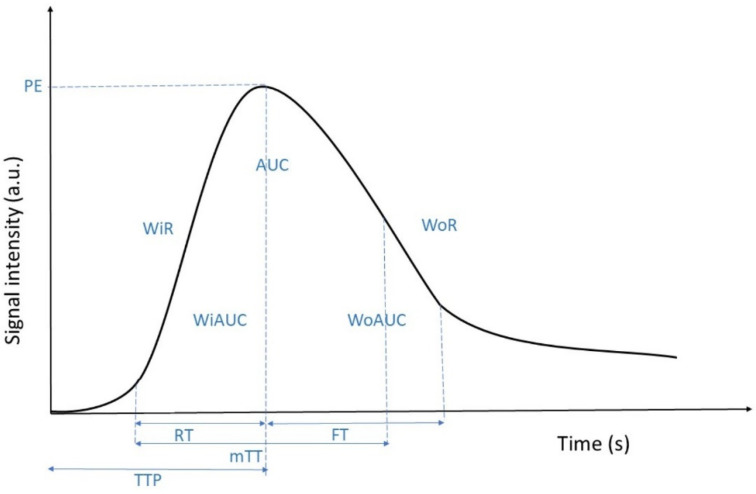
Schematic time intensity curve with illustration of the blood flow parameters. Peak enhancement (PE), wash-in area under the curve (WiAUC), wash-out area under the curve (WoAUC)*,* total area under the curve (AUC)*,* rise time (RT), mean transit time (mTTI), time to peak (TTP), wash-in rate (WiR), fall time (FT), and wash-out rate (WoR).

Seven ROIs were manually drawn: 1 on an interlobar artery, 3 in the renal cortex, 2 in the renal medulla, and 1 surrounding the entire kidney (Figure 1). The ROIs in the medulla were identical in size and shape and drawn at approximately the same depth. The size of the ROIs in the cortex could be adapted only when absolutely necessary (e.g., when the size exceeded the limit of the cortex) and were drawn at approximately the same depth. For every ROI, the software determined mean pixel intensities and created a time–intensity curve. Time–intensity curves were analyzed for blood flow parameters representing blood volume (peak enhancement (PE), wash-in area under the curve (WiAUC), wash-out area under the curve (WoAUC), and total area under the curve (AUC)) and blood velocity (rise time (RT), mean transit time (mTTI), time to peak (TTP), wash-in rate (WiR), wash-in perfusion index (WiPI), fall time (FT), wash-out rate (WoR)) (Figure 2). The values for the 3 ROIs for the cortex and 2 ROIs for the medulla were averaged. Peak enhancement and WiAUC for the cortex, medulla, and entire kidney were normalized to values from the interlobar artery (PE* and WiAUC*).

Two separate random effects models were fitted to assess the variation due to observer on the one hand, and due to the period on the other hand. In a first model, the period was fixed and within a particular period and location combination, the variance components due to sample (*S_s_*^2^), observer (*S_o_*^2^), interaction between observer and sample (*S_oxs_*^2^), and residual variation (*S*^2^) were estimated. Using these variance components estimates, the inter- and intraclass correlation coefficients were calculated. They were defined as

$$ICC_{inter}=\frac{S_{s}^{2}}{S_{s}^{2}+S_{o}^{2}+S_{oxs}^{2}+S_{}^{2}}$$

$$ICC_{intra}=\frac{S_{s}^{2}+S_{o}^{2}+S_{oxs}^{2}}{S_{s}^{2}+S_{o}^{2}+S_{oxs}^{2}+S_{}^{2}}$$

Values close to 0 means very low reliability, 1 means excellent reliability.

In the second model, the observer was fixed and within a particular observer and location combination, the variance components due to sample (*S_s_*^2^), period (*S_p_*^2^), interaction between period and sample (*S_pxs_*^2^), and residual variation (*S*^2^) were estimated. Using these variance components estimates, the inter- and intraclass correlation coefficients (ICC_inter_ and ICC_intra_) were calculated. They are defined as

$$ICC_{inter}=\frac{S_{s}^{2}}{S_{s}^{2}+S_{p}^{2}+S_{pxs}^{2}+S_{}^{2}}$$

$$ICC_{intra}=\frac{S_{s}^{2}+S_{p}^{2}+S_{pxs}^{2}}{S_{s}^{2}+S_{p}^{2}+S_{pxs}^{2}+S_{}^{2}}$$

Values close to 0 means very low reliability, 1 means excellent reliability.

Based on the guidelines from Koo and Li (2016), agreement was considered poor if ICC was less than 0.50, moderate if it was between 0.50 and 0.75, good if it was between 0.75 and 0.90, and excellent if it was greater than 0.90 [23].

## 3. Results

### 3.1. Intra- and Inter-Observer Variability

The mean intra- and interclass correlation coefficients of the renal perfusion parameters are outlined in Table 1. Most renal blood flow parameters had an ICC_inter_ very similar to their ICC_intra_. For FT, PE, WiPI, WiR, and WoR of the entire kidney; FT, PE, WiAUC, WiWoAUC, WiR, and WoR at the medulla; FT, RT, and TTP at the cortex and TTP at the renal artery, ICC_inter_ was considerably lower than ICC_intra_, indicating that changing the observer makes a substantial difference. At the entire kidney, ICC was significantly lower than in other regions of the kidney. The parameters with the best mean ICC_intra_ and ICC_inter_ in general were TTP at cortex, medulla, and entire kidney, mTTI and WoAUC at the artery and cortex, PE, WiPI, WiAUC at the artery, cortex, and medulla and WiWoAUC at the artery, cortex, and medulla. The mean ICC of these parameters over the 3 periods was greater than 0.75 for both the intra- and inter-observer agreement at the respective locations.

In general, the first and second most common source of variation of all blood flow parameters were sample and residual variation, respectively. Occasionally, a considerable part of the variation was attributable to observer sample interaction. Parameters for which a considerable portion of variance was attributable to observer sample interaction were TTP at the interlobar artery and cortex; WiAUC at the interlobar artery and medulla; WoR at the medulla and entire kidney; WiPI, WiWoAUC, and mTTI at the medulla. The only blood flow parameter where a large proportion of the variation was due to the observer was PE*, more specifically at the interlobar artery and cortex.

### 3.2. Intra- and Inter- Periodic Variability

Results concerning within-cat variability in the short-term were previously published by Stock et al. [21]. Briefly, relatively little variation was observed for time-derived parameters, while moderate-to-high variation was found for intensity-related parameters and parameters related to the slope of the time-intensity curve. 

Mean intra- and inter-period agreement for measurement of the renal perfusion parameters are summarized in Table 2. The renal perfusion parameters showed a varying ICC depending on the area within the kidney. A significantly lower ICC was found when the ROI encompassed the entire kidney than at other locations of the kidney. 

Overall, intra-period agreement was markedly better than inter-period agreement for all blood flow parameters. The blood velocity parameters mTTI at the cortex and TTP at the entire kidney were the parameters with the best ICC_intra_ and ICC_inter_. 

For PE, WiPI, WiR, and WoR at the artery, variation between periods was sometimes so large that the ICC_inter_ was 0. This means that if these assessments of different periods would be used, the reliability would be very low, estimated as zero. On the other hand, the ICC_intra_ was often high, this corresponds to the variation between measurements on the same sample within the same period.

No clear trend could be observed in the sources of variation within the blood flow parameters or locations of periodic variability. Variation due to sample, period, sample period interaction, and residual variation were all a common source of variation.

## 4. Discussion

Contrast-enhanced ultrasound (CEUS) enables quantitative assessment of microvascular tissue perfusion and offers numerous advantages over existing imaging modalities such as CT and MRI. Despite its advantages, one of the greatest challenges of CEUS remains its significant variability in tissue perfusion quantification, which complicates diagnostic applications [15]. To date, various sources of variability have been identified, such as operators, scanner settings, contrast agents, and patient-inherent factors [15]. However, an additional source of variation might be introduced due to selection of ROIs. Delineation of ROIs is a manual process based on an observer’s assessment of the anatomic configuration. As this assessment may differ between observers and within the same observer at different occasions, ROI delineation is subject to intra- and inter-observer variability. Furthermore, this process is often complicated by image artifacts, similar signal intensities between the target area and surrounding tissue, and anatomical variations [24]. Therefore, this study aimed to assess intra- and inter-observer variability for quantification of renal perfusion using contrast agent bolus injection.

Intra- and inter-observer variation can be minimized by adherence to a standardized procedure in which operator dependent factors are controlled. However, no consensus guidelines for renal perfusion quantification by CEUS are available in veterinary medicine. Therefore, divergent CEUS protocols can be found in literature, especially regarding ROI selection. In particular, the number, size, and placement of the ROIs drawn in the different renal areas varies between studies [13,14,18,21,25,26,27]. In some studies, the ROI covered the entire cortex or medulla as well as possible [14,26,28]. In others, several smaller ROIs were drawn per renal area and their values were averaged [13,18]. One study used one larger ROI that did not fully enclose the cortex or medulla, rather than averaging multiple smaller ROIs [25]. However, each protocol seems to have a downside. Protocols in which the ROI does not encompass the entire cortex, medulla, or kidney still leave scope for arbitrary ROI placement, while the risk of inclusion of neighboring tissue and large blood vessels increases when the ROI covers an entire renal region.

Although most renal blood flow parameters in this study showed a very similar intra- and inter-observer agreement, several renal perfusion parameters had a considerably better ICC_intra_ than ICC_inter_. When the ROI covered the entire kidney or the medulla, more parameters had a lower ICC_inter_ than ICC_intra_ than when the ROI only covered the cortex or artery. Therefore, different observers appear to cause some variation in perfusion quantification, especially if the ROI surrounds the entire kidney or the medulla. The guidelines on ROI placement in this study may have limited the variation between observers and the influence of local blood flow heterogeneities, but ROI placement remains a manual action allowing for introduction of variability. Another possible explanation for the lower reliability between observers than within observers may be the different level of expertise. Neighboring tissue incorrectly included in the ROI can affect the TIC and associated parameters, especially if the ROI has to encompass a small area such as the interlobar artery [29]. A study investigating intra- and inter-observer variability of echocardiographic measurements in cats found an effect of observer experience indicating that the most competent observer should not to be replaced by a less competent observer [30]. In addition, Khoo et al. (2016) reported a reduction in intra- and inter-observer contouring variations of the prostate in humans after observers followed an education program [31]. 

Solely based on the intra-observer agreement, the results of this study suggest that the cortex is slightly better suited for renal perfusion assessment. The mean ICC of all parameters in the cortex was good, in contrast to the other renal regions where multiple velocity-related parameters showed a poor or moderate mean ICC. This result may be explained by the fact that the cortex has a homogeneous perfusion [27,32]. However, despite the heterogeneous blood flow in the medulla caused by the vascular architecture and physiological characteristics of the medullary blood supply, a considerable number of parameters showed a good intra-observer agreement [33,34]. This may be related to the more stringent guidelines on the ROI placement in the medulla which allowed less influence by the observer. At the level of the medulla and interlobar artery, intensity-related parameters and TTP also seem suitable for renal perfusion assessment. When the ROI encompassed the entire kidney, only TTP showed an acceptable intra-observer agreement. This result is contrary to that of previous studies in humans, rats, and dogs [18,26,35]. These studies reported good to excellent intra-observer agreement for the intensity-related parameters and the velocity-related parameters in both cortex and medulla. A study of Yoon et al. (2020), on the other hand, showed excellent intra-observer agreement for WiR and poor agreement for RT in the medulla and cortex [36]. Other parameters showed moderate intra-observer agreement at both locations [36].

Between observers, a good agreement was demonstrated for intensity-related parameters at the cortex, medulla, and interlobular artery. With the exception of TTP at the cortex, medulla, and entire kidney and mTTI at the renal artery and cortex, time-related and slope-related parameters showed poor agreement among observers. These results are in line with those of other studies. Kay et al. (2009) who studied renal perfusion in the post-transplant period found a good agreement for TTP, PE, WiR, and WiAUC at the level of both the cortex and medulla. Except for WiAUC, these researchers also observed good agreement for interlobar artery perfusion parameters. Two other studies which assessed inter-observer agreement of renal cortical perfusion in human patients reported a good reproducibility for intensity-related parameters and the velocity-related parameters mTTI and TTP [28,37]. Observers in these studies were also bound by guidelines on ROI placement, although these differed from the current study. Kay et al. (2009) limited analysis to either the upper or mid-pole measurements [18]. Schneider et al. (2013) ensured that a similar portion of the renal cortex was examined by applying anatomical landmarks and visual comparison [28]. In addition, all visible superficial cortex closest to the ultrasound probe was enclosed within the ROI [28]. Unlike the current study, however, all observers in the aforementioned studies had extensive experience with CEUS. In the studies of Schneider et al. (2013) and Lin et al. (2022) observers consisted out of well-trained physicians with multiple years of experience. Despite the difference in experience between observers in the current study, the applied ROI placement guidelines may have been sufficient to limit the variation in ROI size, location, and depth to a large extent. Size, location, and depth of ROIs are factors known to affect the TICsand the perfusion parameters derived from them [15,16,17,18,19,20]. In this way the guidelines may have resulted in a good observer agreement for a considerable number of parameters in the current study. 

As indicated by the generally poor ICC_inter_, blood flow parameters of the ROI surrounding the entire kidney proved difficult to reproduce between observers. The only exception was TTP. The reason why blood flow parameters at the entire kidney have a low agreement between observers is unclear. The delineation of the kidney may have been unclear at times, making ROI placement more difficult for the less experienced observer.

Sample and residual variance accounted for the most variability in observer agreement. Sample variance is the part of the variation in the data that is caused by the images being evaluated, while observer variance is the part of the variation in the data that was due to observers [38]. Residual variance is the variability in measurements caused by the influence of variables other than those included in the model [38]. If there are many uncontrolled variables influencing the measurements, in addition to observer and sample, the residual variance can become excessive [38]. When the residual variance is large, it will result in a lower ICC even if observer variance is small [38]. For some parameters, a considerable portion of variance was attributable to observer–sample interaction. Observer–sample interaction can be thought of as the part of the observer effect that may be caused by a specific sample being analyzed. If observer–sample interaction is present, the difference in measurements recorded between observers changes depending on the sample being assessed [38]. Observer–sample interaction reduces intra- and inter-observer reliability [38].

Previous studies in dogs and cats have indicated that TTP, RT, and FT at the cortex and TTP at the medulla are promising parameters for the evaluation of renal perfusion in future research [21,25]. Over the short and long term, these time-related parameters showed the least variation within and between subjects, and therefore may have a greater diagnostic potential [21,25]. Despite that, differentiation between healthy subjects and subjects with renal disorders has also been achieved with blood volume parameters and parameters related to the slope of the TIC [10,11,12,14].

A possible limitation in this study which could have affected the inter-period agreement is that each kidney of each cat was at least evaluated once. As a result, a different kidney was evaluated at one of the three time points, which may have led to additional variation. It is, however, very unlikely that this would have significantly affected the inter-period agreement. Stock et al. (2018) and Liu et al. (2019) namely found that the right and left kidney of the same individual contributed very little to the variation of all perfusion parameters [21,25]. A study of Lin et al. (2022) on diabetic nephropathy in human patients only reported a slight difference in inter-observer agreement between parameters of the right and left kidney [37]. Another limitation of the study is the number of animals used in the experiment, especially in terms of interobserver reliability.

## 5. Conclusions

In conclusion, most renal blood flow parameters in this study showed a similar intra-and inter-observer agreement; however, several quantitative renal perfusion parameters of CEUS showed a better ICC_intra_ than ICC_inter_. Therefore, it may be advised against having the quantitative assessment of renal perfusion performed by different observers, especially if the observers have different levels of experience. Mandatory certification of ultrasonologists based on system-specific expertise may need to be considered. The intensity-related parameter mTTI at the level of the cortex appears to be the most appropriate parameter for renal perfusion assessment as it shows both a favorable inter-observer agreement and inter-period agreement.

## Figures and Tables

**Table 1:** Mean intra- and inter-observer agreement for measurement of contrast-enhanced renal perfusion parameters.

Variable	ICC	Artery	Cortex	Medulla	Kidney
FT	Intra	0.44	**0.82**	0.63	0.39
	Inter	0.42	0.68	0.31	0.23
RT	Intra	0.54	**0.85**	0.66	0.34
	Inter	0.52	0.72	0.50	0.26
TTP	Intra	**0.87**	**0.89**	**0.79**	**0.82**
	Inter	0.70	**0.75**	**0.77**	**0.82**
mTTI	Intra	**0.84**	**0.82**	0.54	0.50
	Inter	**0.76**	**0.79**	0.44	0.42
PE	Intra	**0.80**	**0.78**	**0.99**	0.66
	Inter	**0.79**	**0.76**	**0.83**	0.43
WiPI	Intra	**0.80**	**0.78**	**0.99**	0.67
	Inter	**0.80**	**0.76**	**0.92**	0.43
WiAUC	Intra	**0.92**	**0.82**	**0.98**	0.63
	Inter	**0.90**	**0.80**	**0.84**	0.54
WiWoAUC	Intra	**0.90**	**0.82**	**0.90**	0.59
	Inter	**0.84**	**0.80**	**0.76**	0.53
WoAUC	Intra	**0.87**	**0.82**	**0.79**	0.56
	Inter	**0.87**	**0.80**	0.74	0.51
WiR	Intra	0.68	**0.76**	**0.75**	0.53
	Inter	0.67	0.69	0.42	0.29
WoR	Intra	0.70	**0.76**	0.69	0.50
	Inter	0.69	0.68	0.37	0.27

Inter- and intra-class correlation coefficients > 0.75 (considered to be good or excellent) are in bold. Fall time (FT), rise time (RT), time to peak (TTP), mean transit time (mTTI), peak enhancement (PE), wash-in perfusion index (WiPI), wash-in area under the curve (WiAUC), total area under the curve (WiWoAUC), wash-out area under the curve (WoAUC), wash-in rate (WiR), and wash-out rate (WoR).

**Table 2:** Mean intra- and inter-period agreement for measurement of contrast-enhanced renal perfusion parameters.

Variable	ICC	Artery	Cortex	Medulla	Kidney
FT	Intra	0.47	**0.85**	0.60	0.37
	Inter	0.01	0.37	0.29	0.18
RT	Intra	0.60	**0.88**	0.59	0.31
	Inter	0.14	0.43	0.38	0.14
TTP	Intra	**0.86**	**0.88**	**0.78**	**0.81**
	Inter	0.51	0.57	0.48	0.73
mTTI	Intra	**0.86**	**0.85**	0.51	0.52
	Inter	0.67	**0.76**	0.09	0.49
PE	Intra	**0.83**	**0.79**	**1.00**	0.60
	Inter	0.00	0.11	0.58	0.21
WiPI	Intra	**0.84**	**0.79**	**0.92**	0.60
	Inter	0.00	0.11	0.54	0.21
WiAUC	Intra	**0.93**	**0.79**	**0.94**	0.54
	Inter	0.14	0.24	0.59	0.23
WiWoAUC	Intra	**0.94**	**0.80**	**0.92**	0.49
	Inter	0.13	0.25	0.49	0.22
WoAUC	Intra	**0.85**	**0.81**	**0.79**	0.44
	Inter	0.09	0.26	0.50	0.21
WiR	Intra	**0.80**	**0.79**	**0.90**	0.29
	Inter	0.00	0.04	0.20	0.24
WoR	Intra	**0.79**	**0.77**	**0.91**	0.26
	Inter	0.00	0.02	0.09	0.23

Inter- and intra-class correlation coefficients > 0.75 (considered to be good or excellent) are in bold. Fall time (FT), rise time (RT), time to peak (TTP), mean transit time (mTTI), peak enhancement (PE), wash-in perfusion index (WiPI), wash-in area under the curve (WiAUC), total area under the curve (WiWoAUC), wash-out area under the curve (WoAUC), wash-in rate (WiR), and wash-out rate (WoR).

## Data Availability

Upon request, the data presented in this study can be provided by the corresponding author.
